# Bibliographical Notices

**Published:** 1851-07

**Authors:** 


### BIBLIOGRAPHICAL NOTICES.

*Illustrations of Syphilitic Disease.* By PHILIP RICORD, D. M. P., &c. &c. *Translated by* THOMAS F. BETTON, M. D., &c.; *with the addition of a History of Syphilis, and a complete Bibliography and Formulary of Remedies, collected and arranged by* PAUL B. GODDARD, M. D., &c. *With Fifty large Quarto Plates, comprising* 117 *beautifully colored Illustrations.* Philadelphia. A. Hart (late Carey & Hart) 126 Chestnut street, 1851. pp. 383, 4to.

"If there is one disease which, more than another, merits the distinction of the term *opprobrium medicorum,* that disease is syphilis." So said a leading British reviewer some thirteen years ago, when the researches and new lights of Ricord were just struggling into notice.

"For little less than three centuries and a half," continues the reviewer, "it has monopolized a share of medical literature nearly as large as all the remaining members of the nosological family put together. And what has been effected by all these labors?" The best answer to this question has long since been again and again afforded, by the various publications of the champion of inoculation and speculum examinations, and by those of his disciples, as well as by the discussions and investigations to which they have given everywhere a powerful impulse. Above all, has it been signally and triumphantly replied to in the famous *Clinique Iconographique de l'Hôpital des Vénériens*, of which the "Illustrations" above announced are the admirable re-print and translation.

In this splendid production of the most celebrated syphilograph of the age, we have not only an ample vindication of our profession against the aspersion of "*opprobrium*," but a striking evidence, at once, of the disheartening nature of the malady itself, as the terror and scourge of evil doers, and of the certainty with which it may be followed and controlled, in many of the phases that formerly served only to puzzle the physician and horrify his patient.

M. Ricord's *Clinique Iconographique* may be regarded as the most complete epitome and practical exemplification of his views and course of investigation, upon all the great questions connected with the study of venereal disorders. Its pictures, whether of the pen or pencil, are drawn with characteristic force and aptness of purpose, from the actual and daily life of his hospital and private practice. It seems to have been his labor of love and the pledge of his professional supremacy, in the speciality to which he has been so long and so efficiently devoted. On all these accounts it would be hailed with acclamation, as the richest offering of its able author. But it is destined to become vastly more popular and influential in every way because it presents in its unequalled lithographic colored plates by far the most beautifully natural and instructive series of demonstrations of syphilitic disease that has yet appeared. As mere specimens of scientific art these representations will vie with the finest essays of Cazenave and Wilson, or of any of the dermatologists or morbid anatomists of the day. It is unnecessary, however, to dwell upon their excellence here; the reputation of the original work is too thoroughly and universally established, to require any further mention of its chief attractions. These have long been operating for themselves, abroad and at home; and we are very sure that the transatlantic prototype is already rapidly winning its way, through similar causes, to a still more widely spread and effectual renown. Many of our readers must have been accustomed to its concise but clear and full descriptions, and life-like and frightfully distinct delineations, as the different numbers issued, seriatim, from the Parisian press; and they will coincide with us in our agreable surprise in finding so excellent an imitation in the American counterpart, presented by the Philadelphia collator and translator.

These gentlemen, and their publishers have earned the thanks of the profession in this country in other ways not less than by the elegant and faithful version of their author's language, and the arrangement and very superior execution of the plates and letter press. They have materially increased the interest of the book, to American readers, by adding to the lessons of M. Ricord, an elaborate and curious history of syphilis, an extensive bibliography, and an extremely useful formulary of remedies. These additions, together with the occasional notes of Dr. Goddard, certainly have enhanced the value of the American edition, so much as to have conferred a double obligation on the American admirers of Ricord; even were the translation and the style of the illustrations less eminently praiseworthy than they unquestionably are.

No one will deny the usefulness of an undertaking which thus brings within the reach of the most contracted means, the best work of its kind in any language. Nor can we doubt the importance of the example thus strikingly afforded to less enterprising editors and publishers. We do not believe that a single venture of this sort has failed to secure an encouraging return for the most liberal outlay of trouble and expense, when these have been properly directed. Indeed, we are so convinced of the eventual success of all these first class publications, apart from their intrinsic value, that we are always glad to have an opportunity to aid them in their progress, and we unaffectedly regret, that in this particular instance, our really earnest wishes in behalf of Drs. Betton and Goddard's admirable offering, have been so unusually long in finding suitable expression.

We cannot undertake a critical analysis, or very detailed notice, but we hope to give some idea of its general scope, and of a few of its prominent and peculiar features. The plates, we are told, "amount to fifty in number, but several are accompanied by two or more cases and illustrations, making the whole number of illustrated cases ninety-two." The originals were drawn and colored from life, under M. Ricord's particular direction, by Messrs. Emile Beau and F. Bion, Parisian artists of the highest standing. And they have been accurately and beautifully copied in this city, under direction of Dr. Goddard, at the establishment of P. S. Duval, and colored after the French by Mr. T. S. Jones.

Some changes have been made in the arrangement of the author, and a few of his plates and cases have been omitted in the American edition. These modifications may be immaterial and even advantageous to American readers, although we are not enlightened on the subject in the preface of the editors. They are none the less unfair to M. Ricord, however, since they necessarily impair the integrity of the production for which alone he holds himself responsible. The fault is venial enough in this case it is true, and it is handsomely atoned for in many ways; still it does appear to us a draw-back, and perhaps the more to be deplored because it stands alone.

In the present melancholy dearth of native literature, we may well be satisfied to make the most of transatlantic skill and learning in any form, within the limits of propriety. The interests of humanity are certainly advanced by such a course, and the circumstances of the country would seem to justify a reasonable use of all the means of improvement that are placed at our disposal, whether they be willingly accorded us or not by the master minds to whom we owe them. But in thus appropriating the intellectual treasures of richer lands than ours, let us at least so wear the borrowed plumes that they shall do no dishonor to their owners. If we must still feed upon the crumbs of European science, do let us have them as they fall,—fresh and free from the smoke and dust of the commentator's kitchen.

To return to our "Illustrations." The first seventy-four pages are occupied with the valuable additions of Drs. Betton and Goddard, which we have already mentioned as bona fide acquisitions, and entitled to high praise; especially as they are printed separately, and are distinctly acknowledged, so as not to interfere with the body of the work.

The introductory portions having been dispatched, we are ushered in medias res, with a long succession of clinical reports and illustrative plates, which offer a most instructive series of lessons upon every thing connected with the different varieties, appearances, stages, localities, complications, treatment,—in a word, the whole history, theoretical and practical, of the subject. He treats, for instance, of primary ulcers, of primary follicular ulcers, primary indurated ulcers, diphtheritic, gangrenous, phagedænic, serpiginous, pultaceous, elevated indurated, annular indurated, indurated granulating ulcers, artificial inoculations, and accidental do.; of primary ulcers, in short, of every shape and kind. Then we have the various complications and accompaning constitutional disorders; such as virulent adenitis or bubo, acute adenitis, indolent ditto, phimosis, eczema of the glans, balanoposthitis, lymphitis, ulcerative adenitis, blenorrhagic urethritis, raspberry-like vegetations with phimosis and perforation of the prepuce, wart-like vegetations, perforating chancre of the frenum, paraphimosis, primary non-indurated ulcer of the upper gum, indurated chancre of the breast, &c. in a nurse, chancres of the anus, &c., &c. We have also an abundant display of syphilides, and of all other secondary symptoms, transition symptoms and tertiary symptoms. This brief and cursory list of topics will afford some idea of the range of clinical demonstration undertaken and successfully prosecuted by this energetic teacher. A glance at the list of contents, as well as the most hasty examination of the numerous figures on the plates, will convince any one of the thorough and comprehensive, and at the same time, strictly practical manner in which he has succeeded in dealing with his revolting theme. A more careful study of the letter press, will bring to the reader's attention another important feature of the book. This is, that M. Ricord in the course of his exposition of the great variety of cases, with the treatment appropriate to each, has managed to illustrate, and at some length to explain, the doctrines and special modes of practice which distinguish the now well established school of which he is the acknowledged head and founder; and which, in some respects at least, are now generally admitted to have had a decided influence upon the prophylactic, as well as therapeutic management of this horrible disease. Plates 2nd and 5th, for example, exhibit three different cases of previously unsuspected chancres on the cervix uteri, and enable him to dwell upon the practical importance of examination by the speculum as the only reliable means of diagnosis in affections of the female sexual organs.

"Analagous cases," says he, "are more common than is supposed, and they are the more dangerous, insomuch that women, so long as the affection remains local and concealed, may thus transmit, in good faith, diseases which men contract, believing that the women with whom they have had connection were not diseased. This, again, is one source of error into which those have fallen who believe in the spontaneous origin of syphilis." Again, in relation to the third case, he remarks, "The suppuration to which they [non-indurated chancres of the neck of the uterus] give rise is abundant, sometimes sanious, may simulate a vaginal discharge, and give rise to a suspicion of blenorrhagia, and subsequently to the transmission of chancres to the male by a woman who had only a discharge." "How many errors of this kind," exclaims our author, "occured before I had made the use of the speculum so familiar in the study of venereal diseases! How many errors are still committed by those who prefer basing their diagnosis on the *story* of the patients, rather than seeking truth with more exactness." The great value of the speculum has long since been fully recognized and acted on; and we are told by the American editor, in a note upon the subject, that it has occurred to him to verify many times the conclusions drawn from these two preceding cases. Examinations by the speculum in doubtful cases have repeatedly resulted in the discovery of chancres very much to the astonishment of the patients who had submitted to the test with a full consciousness of their integrity, notwithstanding the disease which they had been suspected of communicating to their male companions.

In plates 1st, 2d, 3d, 4th, 5th, 6th, (the last two being incorrectly marked) 7th, (not marked at all), and 11th, (likewise not marked), we have, along with other matters of interest, distinct representations of various forms and stages of artificial and accidental inoculation, which forcibly remind us of M. Ricord's greatest triumph in the study of syphilitic diseases.

Plate 9th is occupied with the appearances of the affected parts in two remarkable cases of urethral chancres accompanied with blenorrhagic discharges, which had been fatal to the patients, and had thus afforded an opportunity for a conclusive demonstration of what he had long insisted on, the positive existence of the *chancres larvés*, or hidden primary sores within the region of ordinary gonorrhœa. These two important cases were made the subject of a special memoir to the Academy of Medicine, which was reported on adversely in a long counter-argument by M. Lagneau and M. Cullerier. This counter-report is quoted by the author, in conjunction with the history of the cases, and with a summary rejoinder and a synopsis of his syphilo-graphic creed. Here then we have within the compass of a few pages the pith and substance of the inoculation question and its dependent corollaries. Let us hear M. Ricord's conclusions, as briefly stated at the close of his reply to M. Lagneau. A few words in advance, however, from M. L.'s report would not be out of place. He admits that M. Ricord, having had his attention strongly directed to the occasional occurrence of specific ulcers in the male urethra, accompanied with a blenorrhagic flow and sometimes apparently masked by a genuine gonorrhœa, was induced to repeat his researches on the genital organs of women, and ascertained by repeated application of the speculum that chancres frequently existed deep in the vagina, and even on the os uteri itself. This fact being once established—and it would no longer seem doubtful—continues the reporter, "it follows naturally that similar ulcerations should be found in the urethra of men laboring under blenorrhagia more frequently than has been supposed." After a further exposé of M. Ricord's course of reasoning, he goes on to say, "Therefore that deep-seated chancres of the male urethra, or chancres hidden and masked under the guise of blenorrhagia alone, according to M. Ricord, constitute the virulent blenorrhagia of authors, was in his opinion, proved by the result of inoculation, by the most exact analogy and the closest logic. Pathological anatomy was still waiting to complete the triumph. His last crowning argument he has to-day brought foward for the second time." Then follows a short sketch of the cases for which we refer our readers to the book itself.

"Our colleague adds," continues the report, "If to these convincing proofs, it be added that the bubo which accompanies a blenorrhagia, the pus or muco-pus of which gives no results by inoculation, furnishes none in its turn when it suppurates; whilst the bubo, by absorption of the chancres, furnishes like the other an inoculable pus, we shall see a notable difference. If on the other hand, that of the secondary symptoms of lues, chancre is the most common and constant precursor, and that blenorrhagic symptoms are rarely followed by constitutional disease, we will find an easy explanation and reason for maintaining opinions apparently discrepant, and yet in fact similar. The truth might be thus expressed: the primary symptoms of lues is chancre, the form and character of which cannot be the same in all the tissues, and which concealed in the mucous membrane, may either simulate a blenorrhagia, or be masked by it. Without a urethral chancre in blenorrhagia, secondary symptoms are impossible. The rarity of chancre in the urethra during a blenorrhagia, because the necessary predisposing causes are rarely found, explains the relative rarity of the true virulent blenorrhagia of authors, or what I call *chancre larvé*, compared with the numerous cases of mild blenorrhagia, and agrees with the observations of all good authors." "These," continues M. Ricord, as quoted by the report, "are propositions of great importance, which have arisen from close observation, and capable undoubtedly of great qualification, but which I have reduced in volume in order not to waste the time of the Academy."

The objections arrayed against his propositions are summed up by M. Ricord thus:

"Urethral chancres are rare. M. Lagneau has seen *mild* and *catarrhal* blenorrhagia followed by all the symptoms of constitutional syphilis. M. Lagneau says that coryza, conjunctivitis, and discharges from the rectum may be syphilitic without the existence of ulcerations, and may be followed by symptoms of constitutional lues, or propagate syphilis. He adds, that when I assert that the pus of chancre may produce non-virulent blenorrhagic discharges like other irritants, it is impossible for me to prove the assertion. To conclude; the last objections are urged against the uncertainty and dangers of inoculation."

These objections are well disposed of in a few words, by M. Ricord, who concludes his answer with his well known propositions, which unless more successfully controverted than they have yet appeared to be in the midst of protracted opposition, certainly "will add some positive results to a branch of knowledge which has hitherto been enveloped in the most absurd and ridiculous mystery." (vide p. 153.)

They are stated as follows:

"I. The syphilitic ulceration, at the specific period, produces an irritant pus, which, by inoculation, gives rise to a characteristic pustule followed by an ulcer identical with the former, and so on.

"II. Whatever may be the apparent qualities of the pus furnished by an inflamed surface, without regard to the stage or degree of inflammation, if there is no *specific* ulcer, the inoculationis nugatory and unproductive.

"III. No ulcer of not well-proved syphilitic origin, or no artificial ulcer simulating syphilis, can furnish inoculable pus.

"IV. There is not a single symptom of primary syphilitic ulcer which may not be found in ulcers without a trace of syphilis, or which may not be artificially produced; the most perfect assemblage of symptoms, belonging to the true Hunterian chancre, furnishing merely a rational diagnosis. The only absolute, unequivocal and pathognomonic sign, which natu r or art cannot imitate, is the character of the secretion demonstrated by the results of inoculation.

"No one more than myself, has shown the inconveniences and dangers of inoculation, when I proved, contrary to many authors, that the pus of chancre, not only necessarily reproduces chancres, but likewise an ulcer identical with the former. If the inoculation is performed on the same person, the varieties of chancre depend on individual condition, and not on a variety in the specific cause, which is always the same.

"I have also proved the danger of inoculation with pus, reputed non-malignant, such as that of buboes, as was first asserted by M. Cullerier, who has since entirely yielded to my opinion.

"Whilst exhibiting all the cases to the numerous pupils attending my clinic, and publishing, consequently, all my researches, I have never concealed the dangers which might arise from the indiscriminate use of inoculation, but I have shown the advantages that practice and science might reasonably hope to derive therefrom.

"Inoculation performed with proper precaution, is now one of the most innocent operations, because we can promptly arrest its effects after having obtained the information desired. Cauterization with Vienna paste, on the fifth day, at which period, all the characters of the pustule and ulcer are sufficiently developed, always succeeds; and frequently the eschar, which results, leaves in falling off, the subjacent tissues already healed; or if it falls off at an earlier state, there remains only a simple wound which cicatrizes rapidly."

We have given this summary in full because of its importance, although the doctrine it contains has long been a familiar thing; and because that doctrine has continued to gain ground and strength from year to year, and has become as little a matter of conjecture and crude experiment, as it is one of novelty.

Want of time and space will oblige us to pass rapidly over the remainder of the volume. We shall refer as briefly as we can to a few of the cases which may suggest some practical remarks.

To go back a little, we may select case 3d, p. 126, (see fig. 4th of plate 6th,) as presenting some points of particular interest, at the same time, that it is a pretty fair specimen of the clinical reporting of the book in general.

"*Pic—23 years of age, by trade a gilder, admitted October 8th*, 1839. From this patient's account, he has long been subject to herpes preputialis, frequently occurring, but generally disappearing without treatment, after a duration of seven or eight days. The disease under which he now labors has existed for three weeks, and was observed three days after sexual connection. The first symptom he perceived was an ulcer on the internal surface of the prepuce, and which now is in the stage of reparation. This was soon followed by other ulcerations, and follicular inoculation.

"At this date, the glans is red, slightly swollen, and presenting on its surface several eczematous vesicles. On the corona glandis and the mucous membrance of the prepuce are numerous follicular ulcers resembling, for the most part, apthæ. The earliest of the ulcers, also the most extensive, has reached the state of reparation, and appears evidently due to the junction of several groups of ulcers. Its roseate base, already projecting beyond the adjacent parts, thus constitute one of the varieties of ulcers designated by the term *ulcus elevatum*. The edges of some of the chancres in the progressive stage are perpendicular, and their gray base formed by an adherent coat. Others still in the incipient stage, having the vesiculo-pustular form, resemble so exactly the eczematous vesicles on the glans that an examination of their apparent characteristics scarcely marks the difference between them.

"In this patient a recollection of the order in which the symptoms appeared, prove to us that the virulent pus furnished by the first ulceration, has become the cause of two very distinct phenomena; on the one hand, by the infection of the follicles in the vicinity of the corona glandis, it has given rise to follicular chancres; and on the other, by its acridity as an irritating substance, it has favored the developement of the eczematous eruption on the glans.

"The thigh of the patient was inoculated with pus from one of the vesicles on the glans, as well as with pus taken from one of the eczematous vesicles developed on the surface of the balanitic mucous membrane.

"The chancres were cauterized with nitrate of silver and dressed with charpie soaked in aromatic wine. The eczema of the glans was treated by a solution of nitrate of silver, (1 part in 250 of distilled water.) Half diet.

12th. "The inoculation with the pus from the corona glandis has produced the pustule characteristic of the primary ulcer; it was torn open, and beneath the skin was found throughout clearly divided by an ulcer with perpendicular sides and grayish base. The inoculation with the pus from the vesicle of the eczema has produced no result, and the distinction between the symptoms, so difficult on mere inspection, has thus been rendered certain by the artificial inoculation. Three-fourths diet.

"29th. The use of the nitrate of silver and aromatic wine has much improved the aspect of the chancres on the corona glandis. The ulcer in the state of reparation of which the base was projecting, has become level with the surrounding parts; and the grayish coat, formed on the chancres in the progressive stage has nearly disappeared. Cleanliness and the lotion of nitrate of silver will remove the eczematous vesicle from the balanitic mucous membrane. Continue the dressings and regimen.

"Nov. 6th. The ulcers are every where in the stage of reparation. They are superficially touched with nitrate of silver. The dressings and regimen are continued; and on the 15th, he was discharged well."

Plate 8th, exhibits a very graphic illustration of a bad case of primary ulcers, acute balano-posthitis, phimosis and gangrene. The practical, point an account of which we quote from its history, is the failure of inoculation, which, in the words of the writer, "proves to a certainty that the gangrene has radically modified the specific nature of the primary ulcers. It is also important to observe," continues he, "that the pus mixed with gangrenous detritus, the contact of which had produced an erythema of the parts adjacent, did not furnish on inoculation, the smallest symptom which it was possible to confound with those resulting from inoculation with the pus containing syphilitic virus, an agent indispensable in developing the regular phenomena always produced by its introduction under the epidermis, and which cannot be simulated by the more or less acrid secretions furnished by the genital organs, whatever may be the degree of inflammation of the ulcers seated on them."

Plate 10th, affords an additional illustration of gangrenous primary ulcer, and of the non-inoculating property of its purulent secretion. Extreme tension of the tissues in this case, and threatened extension of the gangrene, rendered it important to remove the phimosis by an operation, which, as M. Ricord remarks, should be resorted to "as rarely as possible during the existence of chancres in the progressive stage, for the virulent pus does not fail to inoculate the edges of the incision, and thus extend the ulceration." The operation was performed, however, as a matter of necessity, and in the hope that the specific character of the primary ulcers whose existence was suspected, had been modified by the gangrene, which had mixed its detritus with the secreted pus. It afterwards appeared that the lips of the incision did not ulcerate, "thus verifying the prognosis given as to the destruction of the virulent principle by gangrene. Nevertheless, in order to establish incontestably this point, some pus from the primary ulcer was inserted into the left thigh, and the right thigh was inoculated with pus taken from the superior angle of the division of the prepuce." These inoculations produced no effect.

On plate 21st, we find a specimen of virulent primary ulcer of the gum, contracted by the application of the mouth to the genital organs of a woman affected with chancre.

This is the only case ever met with by M. Ricord, although he tells us that he has several times seen chancres of the lips and tongue. His "object in adducing this example, was to prove that the inoculable virulent syphilitic pus does not select any particular organ, and that it always acts locally, wherever the conditions necessary for its development are found." "Moreover, as a proof that the severity of syphilis does not depend, as has been said, on the location of the primary disease," we are informed, "that this patient had no constitutional symptoms, for he remained healthy long after the period [within six months, if no mercury has been taken or employed,] at which they should have occurred."

The plate (No. 24) and cases to which we next refer, introduce us to "one of the most diffiult problems of syphilitic pathology." The transmission of syphilis from the child to the nurse, and *vice versa*. The two cases are respectively that of a nurse and child, both affected with secondary symptoms, and the nurse also with primary—the child having had a syphilitic father; and that of a child of a healthy mother and healthy *husband*, but of whom the *real father* was secondarily diseased. They show the difficulty, easily imagined, of getting at the truth, so far as parents and nurses are concerned to conceal and misrepresent it.

In regard to contagion from secondary affections, M. Ricord says that he has frequently met with cases favorable to this belief. But whenever he has seen the infant and nurse in the commencement, which often occurs, he has been always able to distinguish the coincidence of secondary symptoms from true contagion produced by the contact of inoculable primary disease (or chancre.)

In relation to the important question of the transmission of syphilis from the parent to the child, he advances the following propositions, based upon a large number of cases.

"I. The father and mother may transmit the disease to their child indifferently, if either or both of them be affected.

II. Transmission may occur from the parents to the child, when they are affected with constitutional symptoms, or when a concealed syphilitic diathesis exists in them.

III. The absence or existence of constitutional symptoms in parents, at the moment of impregnation and conception, exerts no influence on the form of the disease which may afterward appear in the child. The distinction established by M. Cazenave between congenital and hereditary syphilis, and which is based on the absence or presence of constitutional symptoms in the parents at the moment of generation, or which have been developed in the mother during gestation, is totally erroneous; and, indeed, M. Cazenave confesses that his opportunities of observing it have not been ample.

IV. The character and period of the manifestation of the symptoms in the child are governed by the stages to which the disease had advanced in the parents at the moment of generation. The treatment to which the parents were subjected, may also retard, prevent, or modify the appearances in the child.

V. If the parents are both healthy at the time of generation, and the mother contracts syphilis during gestation, she may transmit the disease to her child. Of this I have seen several examples, at various periods of pregnancy, even to the seventh month inclusive.

VI. When the venereal poison is transmitted from the mother to the child during pregnancy, infection takes place through the medium of the placenta, and, in this case, appears to occur after the fourth month of utero-gestation.

In abortions which are consequent on syphilis, if the father alone be diseased at the moment of generation, the abortion may occur at any period of pregnancy. If the mother alone be diseased at the time of conception, the abortion will not take place until after the fourth month.

VII. Children born of a father or mother affected with syphilis may escape infection; for a certain disposition to receive constitutional disease is necessary for the child as well as the adults, and this may be absent.

VIII. Observations made as accurately as possible, seem to prove that constitutional syphilis may be trasmitted from the child to the mother during utero-gestation."

In connection with this subject, we may cite a case of bad tertiary affection of the throat, (see p. 278,) shown in fig. 3d, plate 30th. "Notwithstanding the doubts which may exist concerning paternity, in spite of the legal precept '*Pater est ille quem nuptiæ demonstrant*,' I think it important here," says our author, "to point out what, in this case, bears on the question of transmission on the part of the father, and which relates to what may be observed in a more rigorous manner, when discussing transmission by inheritance from the mother to the child. Experience teaches us that in the stage of tertiary symptoms, parents may give birth to children presenting no trace of syphilis."

"At the time of his marriage in 1845, M. C—— had no syphilitic symptom, and in thirteen months his wife was delivered of a perfectly healthy child. This child still remains well;—nevertheless, M. C—— was under the influence of a syphilitic diathesis, for the first deep ulceration of the throat appeared fifteen months after his wife's first accouchement, and, a remarkable circumstance, eleven months before the last recurrence of the tertiary ulcers in his throat, he had a second child, like the first perfectly healthy. Madame C—— has always enjoyed good health."

With these quotations, we are reluctantly obliged to close. We would be glad to avail ourselves of many others of the cases, and the plates accompanying them, for the purpose of calling the attention of our readers to the various matters of doctrine and experience they may bear upon, but must content ourselves with with earnestly recommending the study of the whole work, as the very best assistant in the diagnosis and management of venereal diseases that has yet been published in this country. Of course it does not supersede the systematic and more elementary works, especially those of M. Ricord himself, and we may add of his able English advocate and coadjutor, Mr. Acton. It constitutes what it alone pretends to be, a splendid clinical illustration of the general facts as they are described in the more didactic essays; and as such it must prove in its American dress a most valuable practical companion and adviser to the advanced students and practitioners of this country. The only evidence of want of care in the preparation of the plates, is the almost continual negligence displayed in the numbering of many of the separate figures as well as of nearly all of the plates themselves. This oversight of the lithographer, however, does not appear to have led to any confusion in the binding, and is therefore not so much to be deplored. Still it is embarrassing to readers, and may become a source of trouble in the binding, and we trust that an early opportunity will be taken to remove the difficulty in the later copies, which will doubtless soon be wanted.

*Lectures on the Eruptive Fevers.* By GEORGE GREGORY, M. D., Fellow of the Royal College of Physicians of London ; Physician to the Small Pox and Vaccination Hospital at Highgate. *First American Edition, with notes and an Appendix, by* H. D. BULKLEY, M. D., Physician of the New York Hospital. New York. S. S. & W. Wood, 1851.

This book comprises a course of lectures on the Eruptive Fevers, delivered by Dr. Gregory in 1843 at St. Thomas's Hospital, London, and annually repeated since that time. It is a very complete manual on the subject, distinguished by great learning and research, an extremely agreeable and lucid style, and, we think, very sound pathological and therapeutic views. The additions of Dr. Bulkley are extensive, and his running comments and appendix greatly enhance the value of the work.

Dr. Gregory makes four grand divisions of cutaneous affections. I. The acute febrile affections bringing life into hazard, which he terms the greater exanthemata, viz.:—Small Pox, Measles, Scarlet Fever, and Erysipelas. II. The acute febrile affections not bringing life into hazard—the lesser exanthemata, which he subdivides into two sections. 1. Vesicular affections, Vaccinia, Varicella, Herpes, and Miliaria; and 2. The simple efflorescences, not leading to fluid effusion, Lichen, Urticaria, Roseola, and Erythema. III. The chronic cutaneous affections of a mild or benignant character, Lepra, Psoriasis, Ichthyosis, Impetigo, Elephantiasis, and Moluscum. IV. Chronic cutaneous affections of a malignant character, such as Cancer, Lupus, and Fungus hæmatodes. The two last divisions, the chronic cutaneous diseases, are not treated of.

The subject of small pox occupies a principal share of the author's attention; and he devotes considerable space to the very interesting and important questions—the identity of small pox and cow pox, and the prophylactic value of vaccination. The conclusions presented in connexion with these points—particularly the latter—will arrest general attention. Dr. Gregory proclaims a distrust in the permanent efficacy of vaccination as a preventive of small pox, which we certainly were not prepared for; and his advocacy of a partial return to inoculation cannot but be received as a remarkable and even startling suggestion.

The identity of small pox and cow pox, is a subject not merely of speculative interest, but of a near practical bearing upon the protective value of vaccination. Jenner was sensibly alive to this; and always strongly advocated the opinion that the two diseases were only modifications of each other. He even advanced the idea that cow pox was the original or parental form, which had been converted by time and modifying circumstances into the more malignant variety, small pox.

This theory is rejected in toto by Dr. Gregory, who maintains that the two affections, instead of being identical or analogous, are antagonistic; and that cow pox, far from being of a variolous, is in fact, of an anti-variolous nature—admitting always, however, that "it alters and modifies the human constitution so as to render some individuals wholly, others partially, and, for a time, unsusceptible of small pox." The discussion of this question is so closely mixed, as we have observed, with the subject of vaccination, that, in the absence of any positively determining facts, opinions are formed with reference to it, according to the views entertained of the efficacy of the agent. The advocates of the theory of entire *identity* frankly admit that a "conviction of the non-identity of the two diseases, would go far to shake, in toto, their belief in the real efficacy of vaccination."

The experiments of Mr. Ceely and others have certainly settled the question, that the inoculation of the matter of small-pox, into the teat or vaginal membrane of the cow, produces the secretion of a fluid identical with that of ordinary cow pox, which, when inserted into the human subject, is followed by a regular vaccine vesicle. This fact has strengthened the belief in the identity of the two affections. But it is met by Dr. Gregory with the argument, that other poisons than the variolous poison develop the vaccinia in the vessels of the cow—as matter from the heals of the horse, when laboring under a disease termed the *grease*. And he infers that "the variolous as well as the equine miasm is converted by the constitution of the cow into the vaccine miasm, in the same way as the vaccine fluid is secreted under several forms of feverish excitement." If the experiment of Ceely proves any thing, it is that small pox is the primary, and cow pox the secondary disorder, and not, as Jenner thought, that cow pox is the parent of small pox. Moreover the non-identity of the two diseases is urged from the fact that they may co-exist in the human subject, progressing side by side to maturity, without the slightest disposition to coalesce or hybridize. And we confess that there appears to us to be very great weight in this objection.

Dr. Gregory's opinions as to the gradual exhaustion of the vaccine protection are strikingly in contrast with the absolute feeling of security, which prevailed on the subject, within the recollection of most of our readers. The protective power of cow pox he thinks *complete*, or nearly so, in childhood, say up to the age of eight years. But puberty and other disturbing causes, as change of climate or severe fevers, notably diminish the vaccine energy, leaving, however, a "portion of virtue still clinging about the system sufficient to preserve life, though not to exhaust susceptibility." The results of his statistical investigations on the subject, amount to this: that small pox, in the unvaccinated, is only five times more fatal, than in those who have previously undergone vaccination. And this announcement is followed by expressions of almost unqualified disbelief in the restorative efficacy of REVACCINATION. It is, indeed, recommended. But we are told, that in the larger number of instances, it will be followed by scarcely appreciable benefit, while it sometimes "occasions considerable local distress, and some amount of constitutional disturbance." That, with these opinions, our author turns to inoculation, as at least an occasional resource, is not surprising.

"The imperfect aid afforded by revaccination suggests the question whether, in adult inoculation (between the ages of twelve and twenty,) we might not find a better mode of testing and improving the security of the vaccinated. Small pox, taken casually after vaccination, proves fatal, as we have seen, at the rate of seven per cent. Inoculated small pox proved fatal, in former times, at the rate of only one fifth per cent., or one in five hundred. As regards the extension of small pox from the practice of inoculation, no real danger need be apprehended. The experience of ten years in England has amply demonstrated that the diffusion of small pox is wholly independent of such artificial propagation. Small pox has been as general in England since inoculation was abolished as it was previously. But it is as yet little known, that small pox may be received into and pass through the system without producing either fever or eruption. I inoculated three of my own children at the respective ages of twelve, thirteen, and fourteen, after successful vaccination in infancy, and the result was as follows :—In two, local affection without fever or eruption. In the third case, local affection without fever, but with papular eruption on the seventh day, not advancing to vesicles. I firmly believe that these children are now, and will remain through life, unsusceptible of small pox. The advantages derived from such a course of procedure are these :—If the child's constitution be under the full vaccine influence, no effect will follow. If the vaccine influence be subsiding or altogether lost, then the small pox will be taken at the period of life (puberty) most favorable for safety, instead of being received (as too often happens under the present system) under circumstances the most unfavorable,—for instance, by mothers at the period of parturition, by young women on the eve of marriage, or by young men at a distance from their friends. The state of the law in England prohibits the practice here ; but I hope it will be tried in other countries, where the judgments of medical men are less fettered."

The American editor has met these views with a very able argument in favor of re-vaccination, which we extract at length.

"We feel that the propriety, if not the duty, of revaccination is now so generally acknowledged as to perhaps render it unnecessary for us to adduce evidence in favor of the practice. But as the views of some on the subject may not be so fully established, and as others may feel interested in an examination of the evidence by which it is supported, we have concluded to devote a short space to it.

Some of the earliest, and at the same time most conclusive testimony in its favor, is furnished by its results in the Wirtemberg, Hanoverian, Bavarian, and especially the Prussian armies. Our limits will not permit us even to give a summary of the figures on which the results are founded, and we can only transfer to our pages some of their most striking features and items.

Revaccination was first commenced systematically in the Prussian armies in the year 1833, after having been practised in the Wirtemberg army and among smaller bodies of men for several years previously, and recommended by several leading practitioners, and has been continued in that and in several other armies, and also among large bodies of civilians, from that time to the present. The following are among the results :

In Wirtemberg but one case of variola occurred in five years among 14,384 revaccinated soldiers, and three only among 26,864 revaccinated civilians.

Not a single case of small pox occurred among those who had been revaccinated in the Prussian army in 1836, 1837, or 1839. But three deaths by this disease occurred in all the military hospitals of Prussia in 1841, and of these, one was in a person not vaccinated on entering the army, because it had been done shortly before; a second in a recruit who had not been revaccinated ; and the third is an officer who had been revaccinated some years before, but without success. In 1834, two deaths are recorded of those who had been revaccinated with effect in the Prussian army, and one in 1843. In 1849, but one case was fatal, and this was in a recruit, vaccinated when a child, and who had not yet been revaccinated.

During an epidemic of small pox in Copenhagen in 1828 to 1330, and also a very severe one in 1832, and another in 1835, not a single instance of variolous or varioloid disease was observed among any who had been revaccinated.

In the Danish army, of those who were successfully revaccinated in 1838, not one was attacked with small pox.

In an epidemic of variola at Heidelburgh in 1843 and 1844, described by Dr. Hœffle, of all those attacked, not a single one had been previously revaccinated, while the vaccinations most successfully made did not protect from the most severe varioloid those older than ten years.

M. Lombard stated, during a late discussion at the Belgian Academy of Medicine, that in the dreadful epidemic of variola which has just desolated Liege, none of those who underwent revaccination took the disease. (*Brit. and For. Med. Chir. Rev. Jan.* 1851.)

Steinbrenner, as the result of extensive investigation of the subject, says, "revaccination is the indispensable complement of the first vaccination, not that it is always necessary, as some pretend who admit the loss of its protective power by time, but because it is necessary in very many cases, and because there is no other means of distinguishing such urgent cases from those in which revaccination is unnecessary." (*Traité sur la Vaccine*, p. 684.) He derives his arguments in favor of revaccination from its effects in the different European armies to which we have already alluded, as well as when performed by various individuals on a smaller scale, of which he presents a long array, and says that, in the absence of every other argument, these results are strongly in its favor, because it is impossible that the process should be so often successful unless the success depended upon a predisposition which exposed the individuals to variola. He also demonstrates the necessity of general revaccination by consideration derived from the too great frequency of vaccinations which are not all protective, or only imperfectly so.

M. Bousquet says, after giving a long list of instances of protection by revaccination without a failure, even in the midst of epidemics, a list of which, he says, he could easily extend, "there has not been an epidemic which has not proved, at the same time, the virtues both of vaccination and of revaccination." (*Nouveau Traité de la Vaccine*—Paris, 1848, p. 506.) He also says (p. 501), "the success of revaccination is at the same time the effect and the proof of the wants of the system"—when it succeeds, it not only proves that the protective power of vaccination is diminished, but it supplies a remedy for this diminution."

The following are the conclusions on this subject of the Committee on Vaccination, of the French Academy, as contained in their report to that body, in February, 1845:—

1. Small pox rarely attacks those who have been vaccinated before the age of ten or twelve, from which age, until thirty or thirty-five, they are particularly liable to small pox.

2. Revaccination is the only known method of distinguishing those vaccinated persons that remain protected, from those that do not.

3. The success of revaccination is not a certain proof that the person in whom it succeeds was liable to contract small pox; it merely establishes a tolerably strong presumption that he was more or less liable to take it.

4. In ordinary periods, revaccination should be practised after fourteen years, but sooner during an epidemic.

Among the conclusions of a report on the subject of vaccination, lately made by a Committee to the Belgian Academy of Medicine, are the following:—

"As the immunity conferred by vaccination is not indefinitely absolute, revaccination, at least for a greater number of individuals, is rationally indicated.

"Experience has proved that a recent revaccination preserves from variola, and varioloid, and that, practised on a sufficient scale, conjointly with vaccination, it constitutes a sure means of arresting the progress of this malady when it appears epidemically.

"It succeeds best in proportion as it is most required, that is the more remote the period since the individual has had variola, or has been vaccinated.

"During the prevalence of an epidemic of variola or varioloid, it is prudent to revaccinate all of those whose first vaccination dates ten years back, and all of those whose first vaccination gives rise to any doubt. (*Brit. and For. Med. Chir. Rev.*, Jan. 1851—from *Gaz. Méd.*)

Tommasini was led by his observations of its results during an epidemic of variola in Italy, to recommend it to his fellow-countrymen, and we might add the names of many highly distinguished of our profession and on both sides of the Atlantic, who concur fully in the importance of the practice, and some who even think it criminal to neglect it.

We cannot but feel, therefore, in view of these facts, that the testimony in favor of re-vaccination is too strong to admit of its neglect. If no other argument could be adduced, the fact that it immediately *arrests the course of epidemics* when they appear, and that its faithful performance has almost entirely banished small pox from some armies in which it had formerly committed great ravages, would seem conclusive in its favor.

It may perhaps be said the results in the European armies to which we have referred speak against the manner in which primary vaccination was performed in these cases, and can hardly, with justice, be advanced in proof of what would have occurred, had the process been perfect in every respect. But allowing this to be true, they may still be adduced as a warning of the danger to which our own population is at least partially exposed; for it cannot but be true, that the process has been not unfrequently performed among us in such a way as to diminish, if not entirely destroy, its protective power. Hence, we need revaccination as a test of security, and as the only one within our reach. And when we consider the almost perfect protection, thus far at least, afforded by revaccination, and how trifling is the operation, we can surely hardly entertain for a moment the idea of resorting to variolous inoculation, either as a test, or for additional security against the failures of vaccination—an operation which is at least occasionally fatal, which subjects those upon whom it is performed to some of the serious results which follow in the train of variola itself, and which cannot be practised without multiplying centres of contagion, and thus aiding the ravages of the very enemy it is intended to combat.

It is proper, however, to state, that the value of this practice is doubted by some, and even its propriety questioned.

Occasional instances of an attack of varioloid after re-vaccination doubtless do occur; but they are so rare as to be thought worthy of record when a single one occurs. Bousquet gives the case of an infant. M. Newman also gives a case, after revaccination by himself. (*Prov. Med. and Surg. Jour.* May 1, 1850.) He considers the practice as a test of the efficacy of the first vaccination, and not a renewal of its influence, which, he contends, can never take place. The occurrence of a regular vaccine vesicle after revaccination he regards as a proof that the individual had not previously been successfully vaccinated.

It is true, as our author remarks, that a talented commission, appointed for the purpose in Paris, reported against the practice of revacccination; but it is equally true that another commission (the one whose conclusions we have quoted), appointed for the same purpose, at a later date, reported in its favor.

As to the age at which its practice should be advised, that of about ten or twelve years, is the one most generally recommended, advice founded on the time at which cases of varioloid have been found to commence occurring—the proportion of cases under ten years of age being very small.

Steinbrenner fixes upon the period of twelve to fifteen years of age as the one most proper for revaccination, and says that, if performed at that time, it will protect for life from variola.

Bousquet says that there is danger from variola from the age of ten or twelve to thirty or thirty-five years, and that the time for revaccination commences from the age of ten or twelve years, that its value increases at fifteen years, and is never greater than between twenty and thirty. In times of epidemic prevalence, it should be practised earlier.

In general terms, it may be said, that the prevalence of variola, the exposure of an individual at particular times, and anxiety felt on the subject at any time, may each afford a reason for performing an operation so trifling in its nature, and attended usually with so little inconvenience, that it had better be submitted to more than once unnecessarily, than neglected, when it might have preserved from an attack of a loathsome disease, and perhaps even saved life itself.

We need hardly add, that even greater care should be taken with the second and subsequent vaccinations than with the first, as we have not the same test of its success as in that case. It should therefore be a rule of practice to repeat the revaccination, if not successful the first time, and even to repeat it several times, in such a case; as a failure to produce the disease might happen from some imperfection in the process, as sometimes occurs in the primary vaccination."

